# Association of silent myocardial infarction on electrocardiogram and coronary artery calcium: The Multi‐Ethnic Study of Atherosclerosis

**DOI:** 10.1111/anec.13081

**Published:** 2023-08-08

**Authors:** Richard Kazibwe, Matthew J. Singleton, Charles A. German, Elsayed Z. Soliman, Gregory L. Burke, Joseph Yeboah

**Affiliations:** ^1^ Section on Hospital Medicine, Department of Internal Medicine Wake Forest School of Medicine Winston‐Salem North Carolina USA; ^2^ Section on Cardiovascular Medicine, Department of Medicine WellSpan Health York Pennsylvania USA; ^3^ Section on Cardiovascular Medicine, Department of Internal Medicine University of Chicago Chicago Illinois USA; ^4^ Epidemiological Cardiology Research Center Wake Forest School of Medicine Winston‐Salem North Carolina USA; ^5^ Section on Cardiovascular Medicine, Department of Internal Medicine Wake Forest School of Medicine Winston‐Salem North Carolina USA; ^6^ Division of Public Health Sciences Wake Forest School of Medicine Winston‐Salem North Carolina USA

**Keywords:** atherosclerotic cardiovascular disease, biomarkers, coronary artery calcium score, risk, silent myocardial infarction

## Abstract

**Background:**

Silent myocardial infarction (SMI) on electrocardiogram (ECG) is associated with atherosclerotic cardiovascular disease, but the relationship between SMI on ECG and coronary artery calcium (CAC) remains poorly understood.

**Objective:**

Characterize the relationship between SMI on ECG and CAC.

**Methods:**

Eligible participants from the Multi‐Ethnic Study of Atherosclerosis study had ECG and CAC scoring at study enrollment (2000–2002). SMI was defined as ECG evidence of myocardial infarction in the absence of a history of clinical cardiovascular disease. CAC was modeled both continuously and categorically. The cross‐sectional relationships between SMI on ECG and CAC were assessed using logistic regression and linear regression.

**Results:**

Among 6705 eligible participants, 178 (2.7%) had baseline SMI. Compared to participants without SMI, those with SMI had higher CAC (median [IQR]: 61.2 [0–261.7] vs. 0 [0–81.5]; *p* < .0001). Participants with SMI were more likely to have non‐zero CAC (74% vs. 49%) and were more likely to have CAC ≥ 100 (40% vs. 23%). In a multivariable‐adjusted logistic model, SMI was associated with higher odds of non‐zero CAC (odds ratio 2.17, 95% CI 1.48–3.20, *p* < .0001) and 51% higher odds of CAC ≥ 100 (odds ratio 1.51, 95% CI 1.06–2.16, *p* = .02).

**Conclusion:**

An incidental finding of SMI on ECG may serve to identify patients who have a higher odds of significant CAC and may benefit from additional risk stratification to further refine their cardiovascular risk. Further exploration of the utility of CAC assessment in this patient population is needed.

## Graphical abstract

An incidental finding of silent myocardial infarction (SMI) on electrocardiogram (ECG) was associated with higher coronary artery calcium (CAC). SMI on an ECG may serve to identify patients who have a higher odds of significant CAC and who may benefit from additional risk stratification to further refine their cardiovascular risk.

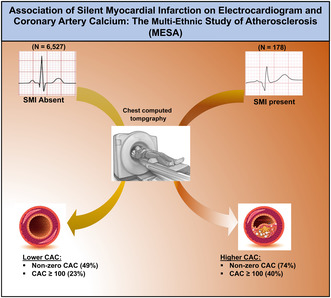

## 1 INTRODUCTION

Silent myocardial infarction (SMI) is defined as electrocardiographic (ECG) evidence of prior myocardial infarction (MI) in the absence of prevalent clinical cardiovascular disease (Soliman, 2019). Prior reports have found that SMI accounts for about half of all MI (Pride et al., 2013). Several studies have noted that SMI is associated with an increased risk of atherosclerotic cardiovascular disease (ASCVD) (Casale et al., 1987; Qureshi et al., 2018; Singleton et al., 2020; Zhang et al., 2016), but the relationship between SMI and coronary artery calcium (CAC) is poorly understood. Therefore, we sought to explore the association of SMI by ECG with CAC in a multiethnic cohort initially free of prevalent clinical cardiovascular disease.

## 2 MATERIALS AND METHODS

### 2.1 Study population

The design and conduct of the Multi‐Ethnic Study of Atherosclerosis (MESA) study have been previously reported (Bild et al., 2002). In brief, MESA is a prospective cohort of 6814 participants, 45–84 years of age, initially free of clinical cardiovascular disease (2000–2002). Participants were recruited from six United States communities: Baltimore, MD; Chicago, IL; Forsyth County, NC; Los Angeles County, CA; New York City, NY; and Saint Paul, MN. For the present analysis, we excluded 109 participants whose baseline ECG could not be assessed for SMI due to either incomplete ECG data (*n* = 104) or competing Minnesota codes that preclude ascertainment of SMI by ECG (*n* = 5), leaving 6705 eligible participants. Study protocols were approved by the institutional review boards at participating institutions. All participants provided written informed consent.

### 2.2 Exposure variables

Silent MI was assessed on baseline ECG and defined as ECG evidence of MI using the Minnesota ECG classification via the presence of a major Q‐wave abnormality (Minnesota Code 1.1.x or 1.2.x) or minor Q/QS waves in the setting of major ST‐T abnormalities (Minnesota Code 1.3.x plus either 4.1.x, 4.2, 5.1, or 5.2) in the absence of history of clinical cardiovascular disease (Prineas et al., 2009).

### 2.3 Outcome variable

The details of the MESA protocols for the measurement of CAC have been reported previously (Carr et al., 2005). Briefly, CAC was assessed by chest computed tomography and referenced to a phantom of known physical calcium concentration. All scans were centrally read at the Los Angeles Biomedical Research Institute at Harbor‐UCLA Medical Center, Torrance, California. The Agatson score was averaged over two scans for all analyses.

### 2.4 Covariates

Baseline covariates were obtained at the initial MESA examination (2000–2002). Current smoking was defined as smoking at least one cigarette in the preceding 30 days. Diabetes mellitus was defined as a fasting glucose concentration ≥126 mg/dL or the use of hypoglycemic medication. Antihypertensive medication use was determined from a review of prescription medications. Electrocardiographic left ventricular hypertrophy was assessed by Novacode criteria (Rautaharju et al., 1998). Self‐reported physical activity was modeled as a categorical variable with possible values of poor (none), intermediate (1–149 min per week of moderate‐intensity or 1–74 min per week of vigorous‐intensity activity or the sum of moderate‐ and vigorous‐intensity activity 1–149 min per week), or ideal (150+ min per week of moderate‐intensity activity or 75+ min of vigorous‐intensity activity or the sum of moderate‐ and vigorous‐intensity activity 150+ min per week).

### 2.5 Statistical methods

Baseline characteristics of the study population stratified by SMI were compared using mean ± standard deviation for continuous variables and frequency (percentage) for categorical variables. Between‐group differences were assessed using analysis of variance for continuous variables and chi‐squared tests for categorical variables. Initially, CAC was modeled as a categorical variable and the odds of elevated CAC using two different thresholds (CAC > 0 and CAC ≥ 100) were compared in participants without and with SMI using logistic regression. Our initial model was unadjusted, with subsequent models adjusted for covariates believed to be of clinical significance or that might confound the relationship between SMI and CAC, including age, sex, and race/ethnicity in Model 2, with Model 3 also adjusting for current smoking, diabetes, systolic blood pressure, antihypertensive medication use, statin use, high‐density lipoprotein cholesterol, total cholesterol, body mass index, left ventricular hypertrophy by electrocardiogram, and self‐reported physical activity. Our secondary analysis considered CAC as a continuous variable and used linear regression to determine the relationship between SMI and CAC. A secondary analysis was performed, in which the CAC score was log‐transformed. The consistency of the above relationships was explored in prespecified subgroups by sex and race/ethnicity by including an interaction term in the model. Two‐sided *p*‐values below .05 were considered to be statistically significant. All statistical analyses were conducted at Wake Forest University School of Medicine using SAS version 9.4.

## 3 RESULTS

Among the 6705 eligible MESA participants (mean age 62.2 ± 10.2 years, 53% women, 38% white, 12% Chinese‐American, 28% African‐American, 22% Hispanic), 6527 (97.3%) did not have SMI at baseline, while 178 (2.7%) had SMI at baseline. Baseline characteristics of the study population stratified by SMI are presented in Table 1. Participants with SMI at baseline were older, more likely to be male, and more likely to have higher systolic and diastolic blood pressures, diabetes or impaired fasting glucose, hypertension requiring antihypertensive therapy, and left ventricular hypertrophy by ECG.

**TABLE 1:** Characteristics of MESA study participants (*n* = 6705) stratified by prevalent SMI.

	No SMI, *n* = 6527 (97.3%)	SMI, *n* = 178 (2.7%)	*p*‐value*
Age (years)	62.1 ± 10.2	66.0 ± 10.3	<.0001
Sex (% male)	3038 (46.5%)	122 (68.5%)	<.0001
Race/Ethnicity			.64
White	2493 (38.2%)	71 (39.9%)	
Chinese‐American	779 (11.9%)	17 (9.6%)	
African‐American	1816 (27.8%)	54 (30.3%)	
Hispanic	1439 (22.0%)	36 (20.2%)	
BMI	28.3 ± 5.5	28.8 ± 5.0	.21
Cholesterol (mg/dL)			
Total	194.3 ± 35.7	189.6 ± 38.2	.09
HDL	51.1 ± 14.8	48.6 ± 15.8	.03
LDL	117.3 ± 31.5	113.3 ± 31.6	.10
Triglycerides	131.4 ± 88.9	136.4 ± 82.5	.46
Blood pressure (mmHg)			
Systolic	126.4 ± 21.4	133.9 ± 24.0	<.0001
Diastolic	71.8 ± 10.2	75.6 ± 10.5	<.0001
Cigarette smoking			.16
Never	3281 (50.4%)	83 (46.9%)	
Former	2368 (36.4%)	76 (42.9%)	
Current	858 (13.2%)	18 (10.2%)	
Physical activity			.20
Poor	1492 (22.9%)	51 (28.8%)	
Intermediate	1157 (17.8%)	28 (15.8%)	
Ideal	3861 (59.3%)	98 (55.4%)	
Diabetes mellitus			.005
Normal	4806 (73.9%)	110 (62.1%)	
Impaired fasting glucose	884 (13.6%)	36 (20.3%)	
Untreated diabetes	170 (2.6%)	4 (2.3%)	
Treated diabetes	644 (9.9%)	27 (15.3%)	
Statin use	957 (14.7%)	33 (18.5%)	.17
Antihypertensive use	2404 (36.8%)	86 (48.3%)	.002
LVH by ECG	57 (0.9%)	11 (6.2%)	<.0001

*Note*: Continuous variables described as mean ± SD. Categorical variables described as frequency (percentage).

Abbreviations: BMI, body mass index; ECG, electrocardiography; HDL, high‐density lipoprotein; LDL, low‐density lipoprotein; LVH, left ventricular hypertrophy; MESA, Multi‐Ethnic Study of Atherosclerosis, SMI, silent myocardial infarction.

* *p*‐value as calculated by ANOVA for continuous and χ^2^ for categorical variables.

**FIGURE 1:**
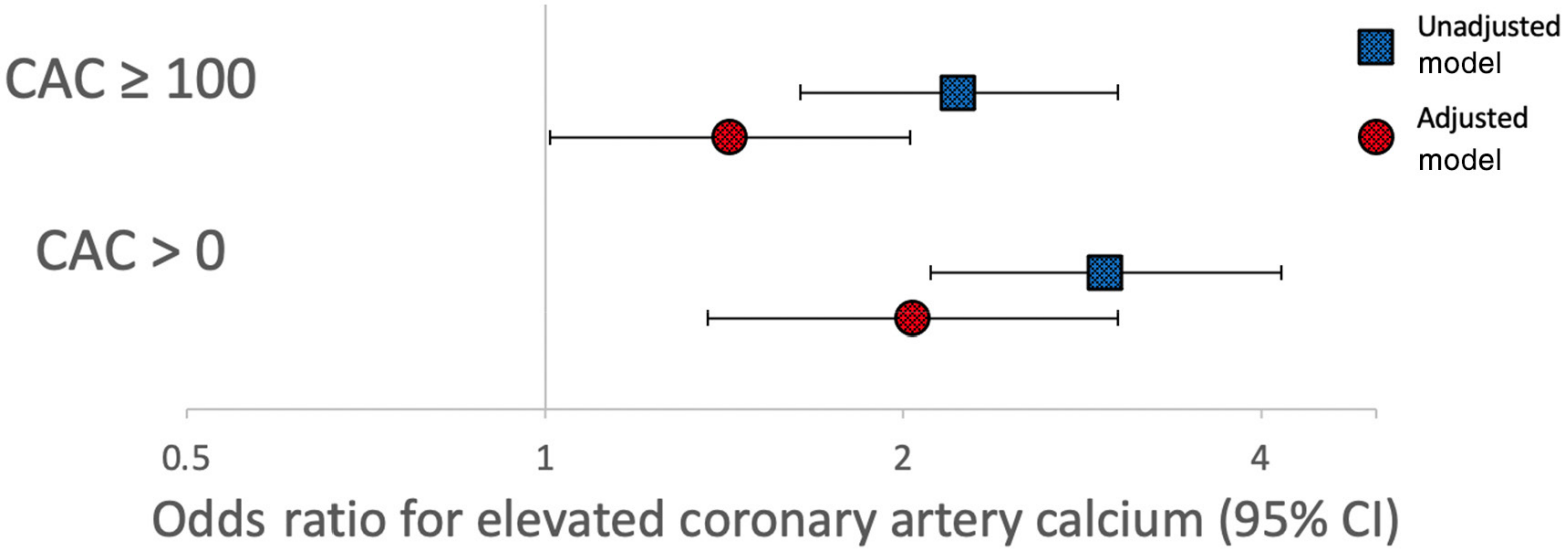
Silent myocardial infarction and coronary artery calcium. Multi‐Ethnic Study of Atherosclerosis participants with silent myocardial infarction had higher odds of non‐zero coronary calcium and coronary calcium greater than 100. Adjusted model includes age, sex, race/ethnicity, smoking, diabetes, systolic blood pressure, antihypertensive medication use, left ventricular hypertrophy by electrocardiogram, and self‐reported physical activity.

Compared to participants without SMI, those with SMI were more likely to have non‐zero CAC (74% vs. 49%) and were more likely to have CAC ≥ 100 (40% vs. 23%; Table 2). In an unadjusted logistic model, SMI was associated with higher odds of non‐zero CAC and higher odds of CAC ≥ 100. After adjusting for demographics and clinical covariates, SMI remained independently associated with higher odds of CAC ≥ 0 and CAC ≥ 100 (Figure 1). When CAC was modeled as a continuous variable, participants with SMI had higher CAC than those without SMI (Table 3). In a multivariable‐adjusted linear regression model, SMI remained independently associated with higher CAC. In light of the lack of normal distribution of CAC, we then log‐transformed the CAC score (natural logarithm of CAC plus 1)—findings were overall similar (Table 4). The above relationships were consistent in prespecified subgroups by sex and race/ethnicity, with *p*‐values for sex‐SMI interaction and *p*‐values for race/ethnicity‐SMI interaction all >.40.

**TABLE 2:** The association between silent myocardial infarction (SMI) and elevated coronary artery calcium (CAC) score using two thresholds (coronary calcium greater than zero or 100 Agatson units) is provided using a multivariable logistic regression model.

	Participants with CAC^a^ above threshold	Model 1	Model 2	Model 3
		Unadjusted odds ratio (95% CI; *p*‐value)	Demographic‐adjusted odds ratio (95% CI; *p*‐value)	Adjusted odds ratio (95% CI; *p*‐value)
**CAC > 0**				
No SMI	3211 (49.2%)	Reference	Reference	Reference
SMI	132 (74.2%)	2.96 (2.11–4.16; <.0001)	2.13 (1.46–3.12; <.0001)	2.17 (1.48–3.20; <.0001)
**CAC ≥ 100**				
No SMI	1496 (22.9%)	Reference	Reference	Reference
SMI	71 (39.9%)	2.23 (1.64–3.03; <.0001)	1.48 (1.04–2.10; .03)	1.51 (1.06–2.16; .02)

*Note*: Silent myocardial infarction is independently associated with a higher odds of non‐zero coronary calcium and coronary calcium greater than 100. Model 1 is unadjusted. Model 2 adjusts for age, sex, and race/ethnicity. Model 3 adjusts for the covariates in Model 2, plus smoking, diabetes, systolic blood pressure, antihypertensive medication use, statin use, high‐density lipoprotein cholesterol, total cholesterol, body mass index, left ventricular hypertrophy by electrocardiogram, and self‐reported physical activity.

^a^ Coronary Artery Calcium (categorical).

**TABLE 3:** The association between silent myocardial infarction and elevated coronary artery calcium score (continuous) is provided using a multivariable linear regression model.

SMI	Participants (*n*)	CAC (mean ± SD)	Model 1	Model 2	Model 3
			Unadjusted *β* (95% CI; *p*‐value)	Demographic‐adjusted *β* (95% CI; *p*‐value)	Adjusted *β* (95% CI; *p*‐value)
No SMI	6527	140.4 ± 409.4	Reference	Reference	Reference
SMI	178	291.5 ± 570	75.6 (44.7–106.4; *p* < .0001)	37.0 (7.7–66.2; *p* = .01)	35.9 (6.4–65.4; *p* = .02)

*Note*: Silent myocardial infarction is associated with a higher coronary artery calcium score in both unadjusted and adjusted models. Model 1 is unadjusted. Model 2 adjusts for age, sex, and race/ethnicity. Model 3 adjusts for the covariates in Model 2, plus smoking, diabetes, systolic blood pressure, antihypertensive medication use, statin use, high‐density lipoprotein cholesterol, total cholesterol, body mass index, left ventricular hypertrophy by electrocardiogram, and self‐reported physical activity.

**TABLE 4:** The association between silent myocardial infarction and elevated coronary artery calcium score (log‐transformed) is provided using a multivariable linear regression model.

SMI	Participants (*n*)	CAC (median, IQR)	Model 1	Model 2	Model 3
			Unadjusted *β* (SE; *p*‐value)	Demographic‐adjusted *β* (SE; *p*‐value)	Adjusted *β* (SE; *p*‐value)
No SMI	6527	0 (0–81.5)	Reference	Reference	Reference
SMI	178	61.2 (0–261.7)	0.67 (0.10; *p* < .0001)	0.32 (0.08; *p* = .0001)	0.31 (0.08; *p* < .0001)

*Note*: Silent myocardial infarction is associated with a higher coronary artery calcium score in both unadjusted and adjusted models. Model 1 is unadjusted. Model 2 adjusts for age, sex, and race/ethnicity. Model 3 adjusts for the covariates in Model 2, plus smoking, diabetes, systolic blood pressure, antihypertensive medication use, statin use, high‐density lipoprotein cholesterol, total cholesterol, body mass index, left ventricular hypertrophy by electrocardiogram, and self‐reported physical activity.

## 4 DISCUSSION

In this cross‐sectional analysis of a contemporary multiethnic United States cohort, we found that SMI on ECG was independently associated with increased odds of elevated CAC.

Since SMI was first described 70 years ago (Hipp et al., 1949), most of the published literature suggests that SMI conveys a worse prognosis and a higher risk of subsequent cardiovascular events (Godsk et al., 2012; Merkler et al., 2019; Qureshi et al., 2018; Stokes & Dawber, 1959). Despite this fact, the way in which an incidental finding of SMI on ECG is incorporated into clinical medicine remains inconsistent. For instance, SMI is not endorsed as a risk enhancer for refining the prediction of ASCVD in the 2018 AHA/ACC cholesterol guidelines (Grundy et al., 2019a), though the presence of a risk enhancer can move the needle toward either initiation of statin therapy or intensification (Arnett et al., 2019). In contrast, CAC has proven value in refining risk (Yeboah et al., 2012) and its use is emphasized in the AHA/ACC guidelines (Grundy et al., 2019a). Our findings suggest that those with incidentally‐found SMI on ECG may represent a patient population with higher odds of elevated CAC. This elevated pretest probability of CAC makes CAC screening more likely to be high‐yield, so consideration of CAC scanning is warranted in patients with SMI by ECG. The use of CAC scanning to refine risk is likely to be particularly valuable in patients at intermediate risk or patients who are reticent to start statin therapy, and risk refinement with CAC scanning received a IIa recommendation in the AHA/ACC guidelines (Grundy et al., 2019b).

The mechanisms of the observed association likely involve both SMI and CAC representing the downstream products of atherosclerosis and preclinical ASCVD. Prior studies have demonstrated that, among asymptomatic patients, diffuse atherosclerosis is highly associated with silent myocardial ischemia (Anand et al., 2004). Similarly, correlates of ASCVD are strongly associated with SMI on ECG, including male sex, advanced age, diabetes mellitus, and renal dysfunction (Arenja et al., 2013; Burgess et al., 2009). Coronary artery calcium is known to be associated with advanced age, obesity, and dyslipidemia (Bild et al., 2001). Together, this suggests that CAC is a marker of subclinical cardiovascular disease and that the association between CAC and SMI may be mediated by atherosclerosis.

It should be noted that, although there is an association between SMI and elevated CAC, a substantial minority of those with SMI had no CAC—25.8% of those with SMI in our study population. This observation is best explained by those with SMI on ECG being a heterogenous group, with some having no significant coronary artery disease. These participants may have SMI due to the fact that there are multiple mechanisms of myocardial infarction, including several that do not include atherosclerosis in the causal pathway, such as non‐ischemic cardiomyopathies, acute myocardial infarctions without non‐obstructive coronary artery disease (Lindahl et al., 2017), and obstructive coronary artery disease that is secondary to lipid‐rich plaque, without associated coronary artery calcification (German et al., 2020). Thus, even though patients with SMI on ECG have an increased odds of elevated CAC, there is likely value in formal CAC scanning, rather than considering all patients with SMI to have presumptively high CAC scores.

Interestingly, while SMI on ECG has been shown to be independently predictive of adverse cardiovascular outcomes (Merkler et al., 2019; Qureshi et al., 2018; Stokes & Dawber, 1959) and improves discrimination in select patient populations (Singleton et al., 2020) but not in other patient populations (Singleton et al., 2021), the correlation of SMI on ECG with the presence and burden of scar on advanced imaging is poor to fair (Dastidar et al., 2016; Kaandorp et al., 2005). It is likely that cardiac magnetic resonance imaging (CMRI) is more sensitive for myocardial scar than ECG, as the quantity of myocardium that must be infarcted before there is ECG evidence of infarction is likely greater than the amount that must be infarcted for detection on CMRI. Prior work from MESA is consistent with this hypothesis, as only 9.3% of participants with myocardial scars detected by CMRI had ECG evidence of MI (Turkbey et al., 2015). However, though CMRI has the benefit of higher sensitivity, ECG is much more affordable and is widely available, so there is value in optimizing the use of ECG for refining risk and choosing the optimal patient population to undergo advanced imaging. Our findings of an association between SMI on ECG and elevated CAC suggest that patients with SMI on ECG may benefit from formal CAC screening to further refine risk.

When stratified by sex and race/ethnicity, the reported relationships between SMI and CAC were conserved, with no evidence of interaction between either sex or race/ethnicity and SMI with regard to CAC. This consistency in the SMI‐CAC association is interesting since prior analyses have found that the prognostic significance of SMI differs by sex and race/ethnicity (Zhang et al., 2016). This consistency of association between SMI and CAC, despite the differential effects on outcomes by sex and race/ethnicity, suggests that some of the risk associated with SMI may not be related to CAC, which is consistent with the literature demonstrating that SMI by ECG and myocardial scar by CMRI are only loosely correlated (Turkbey et al., 2015).

Our study should be interpreted in the context of its limitations. Although we adjusted for covariates with either known or suspected relationships with SMI and CAC, residual confounding always remains a possibility. Our study population was free of known clinical cardiovascular disease at baseline, so our findings may not apply to a higher‐risk patient population with prevalent cardiovascular disease. As the MESA study only included white, Chinese‐American, African‐American, and Hispanic participants, our findings should not be extended to other racial and ethnic groups, though findings were similar in the included race/ethnic groups in our study and there was no evidence of effect modification by race/ethnicity. Advanced imaging, when available, is likely both more sensitive and more specific than ECG for detecting prior infarction, so SMI by ECG is more useful as a screening test and may have little additive value once advanced imaging has been obtained. Finally, study ECG and study CT did not occur simultaneously, so there may have been interval cardiovascular events between the ECG and a given participant's study CT. Strengths of our study include the multiethnic cohort, centralized ECG reading center, and consistent CT imaging protocols used for all participants.

## 5 CONCLUSION

In this analysis of the MESA study, we found that SMI on ECG is associated with higher CAC scores and higher odds of a non‐zero CAC score. An incidental finding of SMI on ECG may serve to identify patients who have a higher likelihood of significant CAC and may benefit from additional risk stratification to further refine their cardiovascular risk—further exploration of the utility of CAC assessment in this patient population is needed.

## AUTHOR CONTRIBUTIONS

Matthew Singleton is the guarantor of this work and, as such, had full access to all the data in the study and takes responsibility for the integrity of the data and the accuracy of the data analysis. All authors contributed to the study's conception and design. Matthew Singleton, Richard Kazibwe, and Joseph Yeboah interpreted the results. Matthew Singleton and Richard Kazibwe drafted the manuscript. Charles German, Elsayed Soliman, Gregory Burke, and Joseph Yeboah revised for critical intellectual content. All authors approved the final manuscript for submission.

## FUNDING INFORMATION

This research was supported by contracts 5N92020D00001, HHSN268201500003I, N01‐HC‐95159, 75N92020D00005, N01‐HC‐95160, 75N92020D00002, N01‐HC‐95161, 75N92020D00003, N01‐HC‐95162, 75N92020D00006, N01‐HC‐95163, 75N92020D00004, N01‐HC‐95164, 75N92020D00007, N01‐HC‐95165, N01‐HC‐95166, N01‐HC‐95167, N01‐HC‐95168 and N01‐HC‐95169 from the National Heart, Lung, and Blood Institute, and by grants UL1‐TR‐000040, UL1‐TR‐001079, and UL1‐TR‐001420 from the National Center for Advancing Translational Sciences (NCATS).

## CONFLICT OF INTEREST STATEMENT

Dr Elsayed Z. Soliman is an Editorial Board member of both Annals of Noninvasive Electrocardiology and CNS Neuroscience and Therapeutics and, a co‐author of this article. To minimize bias, they were excluded from all editorial decision‐making related to the acceptance of this article for publication.

## ETHICS STATEMENT

Study protocols were approved by the institutional review boards at participating institutions. All participants provided written informed consent (Bild et al., 2002).

## Data Availability

The data that support the findings of this study are openly available at National Heart, Lung and Blood Institute Biologic Specimen and Data Repository https://biolincc.nhlbi.nih.gov/studies/mesa/, Ref No. HLB00640822b.
